# Attenuated Lymphatic Proliferation Ameliorates Diabetic Nephropathy and High-Fat Diet-Induced Renal Lipotoxicity

**DOI:** 10.1038/s41598-018-38250-7

**Published:** 2019-02-13

**Authors:** Yaeni Kim, Seun Deuk Hwang, Ji Hee Lim, Min Young Kim, Eun Nim Kim, Bum Soon Choi, Yong-Soo Kim, Hye Won Kim, Cheol Whee Park

**Affiliations:** 1 Division of Nephrology, Department of Internal Medicine, College of Medicine, The Catholic University of Korea, Seoul, Republic of Korea; 2 Division of Nephrology, Department of Internal Medicine, College of Medicine, Inha University, Incheon, Republic of Korea; 3 Institute for Aging and Metabolic Diseases, College of Medicine, The Catholic University of Korea, Seoul, Republic of Korea; 4 Department of Rehabilitation Medicine, College of Medicine, The Catholic University of Korea, Seoul, Republic of Korea

## Abstract

Lymphangiogenesis occurs in response to renal injury and is correlated with interstitial fibrosis. Diabetes- and high-fat diet (HFD)-induced intrarenal lipotoxicity and their relationships with lymphangiogenesis are not established. We used PPARα agonist, fenofibrate, to unravel the linkage between lipotoxicity and lymphangiogenesis. Eight-week-old male C57BLKS/J *db/db* mice and HFD Spontaneously hypertensive rats (SHRs) were fed fenofibrate for 12 weeks. HK-2 and RAW264.7 cells were used to investigate their lymphangiogenic capacity in relation to lipotoxicity. Fenofibrate improved intrarenal lipotoxicity by increasing expression of PPARα and phosphorylation of AMPK. Lymphatic proliferation was attenuated; expression of lymphatic endothelial hyaluronan receptor-1 (LYVE-1), podoplanin, vascular endothelial growth factor-C (VEGF-C), and vascular endothelial growth factor receptor-3 (VEGFR-3) was decreased. In parallel, extent of tubulointerstitial fibrosis, apoptosis and inflammatory cell infiltration was reduced. In HK2 cells, palmitate- and high glucose-induced over expression of lymphatic makers was diminished by fenofibrate via activation of PPARα-AMPK-pACC signaling. Enhanced expression of M1 phenotype in RAW264.7 cells correlated with increased lymphatic growth. A causal relationship between lipotoxicity and lymphatic proliferation with a cellular link to macrophage activation can be speculated; pro-inflammatory M1 type macrophage is involved in the development of lymphangiogenesis through stimulation of VEGF-C and by its transdifferentiation into lymphatic endothelial cells.

## Introduction

While traditional researches deemed the lymphatic vasculature merely as a passive channel that transported various macromolecules from the interstitial space into the blood circulation, its active role in the regulation of tissue fluid homeostasis, immune cell trafficking, and dietary fat absorption has been recently enlightened^1^. Inflammation is frequently linked with profound lymphangiogenesis and lymphatic vessel remodeling, such that increased demand for lymphatic drainage is required to promote swift removal of inflammatory cells, toxic antigens, cytokines, and cellular debris to undo consecutive noxious events that would otherwise lead to chronic tissue damage, including fibrosis^2^. The role of lymphatic vessels in the pathogenesis of diabetic nephropathy (DN) and high-fat diet-induced renal damage has been questioned owing to the development of lymphatic endothelial cell (LEC)-specific markers that allow the visualization of these transparent vessels. Diabetic mouse models show increased distribution of lymphatic vessels in the cortex and medulla, which would otherwise have engaged lymphangiogenesis in the renal cortical area only^3,4^. Lymphatic proliferation is coexistant with areas of tubulointerstitial fibrosis and inflammatory cell infiltration in DN. This pro-inflammatory condition is ascribable to systemic hyperglycemia and intrarenal lipotoxicity that promote increased production of TGF-β and recruitment of macrophages, which coordinately augment the production of vascular endothelial growth factor (VEGF), possibly triggering a cytokine cascade to induce lymphangiogenesis in renal cells^5^.

Lipotoxicity refers to the state of energy surplus in which toxic lipid intermediates accumulate as a consequence of decreased fatty acid β-oxidation and increased fatty acid synthesis, and resultant increase in oxidative stress causing toxicity and cell death within non-adipose organs, including diabetic kidneys^6^. These toxic lipid metabolites and deranged lipid metabolism modulate the expression of macrophage phenotype in such that pro-inflammatory and pro-apoptotic properties are enhanced^7^. A novel finding that peripheral cholesterol metabolites are cleared through lymphatic drainage established a mutual relationship between lipid metabolism and lymphatic function^8^. Moreover, it was recently demonstrated that lymphatic vessels are primarily involved in this efflux of cholesterol, such that restoration of lymphatic structure by VEGF-C administration to apolipoprotein E-deficient (APO-E (−/−)) mice not only improved lymphatic function but also decreased cholesterol content in tissues, independently of changes in the systemic lipid profile.

Given the emerging significance of lymphatic vessels in lipid metabolism, we aimed to investigate the relationship between intrarenal lipotoxicity and dysfunctional lymphatic proliferation, with emphasis on the role of proximal tubular epithelial cells (PTECs) and macrophages as a cellular link that modulates lymphatic remodeling. Fenofibrate is a lipid-lowering agent that acts via the activation of peroxisome proliferator-activated receptor α (PPARα)^9^. We previously reported its potential as a therapeutic means to ameliorate renal lipotoxicity in diabetic mice^10^ and HFD SHRs^11^ via the activation of the AMP-activated protein kinase (AMPK)-Peroxisome proliferator-activated receptor γ co-activator 1α (PGC-1α)-Estrogen-related receptor (ERR)-1α-class O forkhead box (FoxO)3a signaling pathway. We hypothesized that fenofibrate treatment would help restore dysfunctional lymphatic vasculature with regard to reduced intrarenal lipotoxicity and inhibited PTECs and macrophage activation, which would ameliorate intrarenal inflammation and fibrosis, resulting in renal phenotypic and functional improvement.

## Results

### Amelioration of intrarenal lipotoxicity reduces intrarenal inflammation

**Figure 1:**
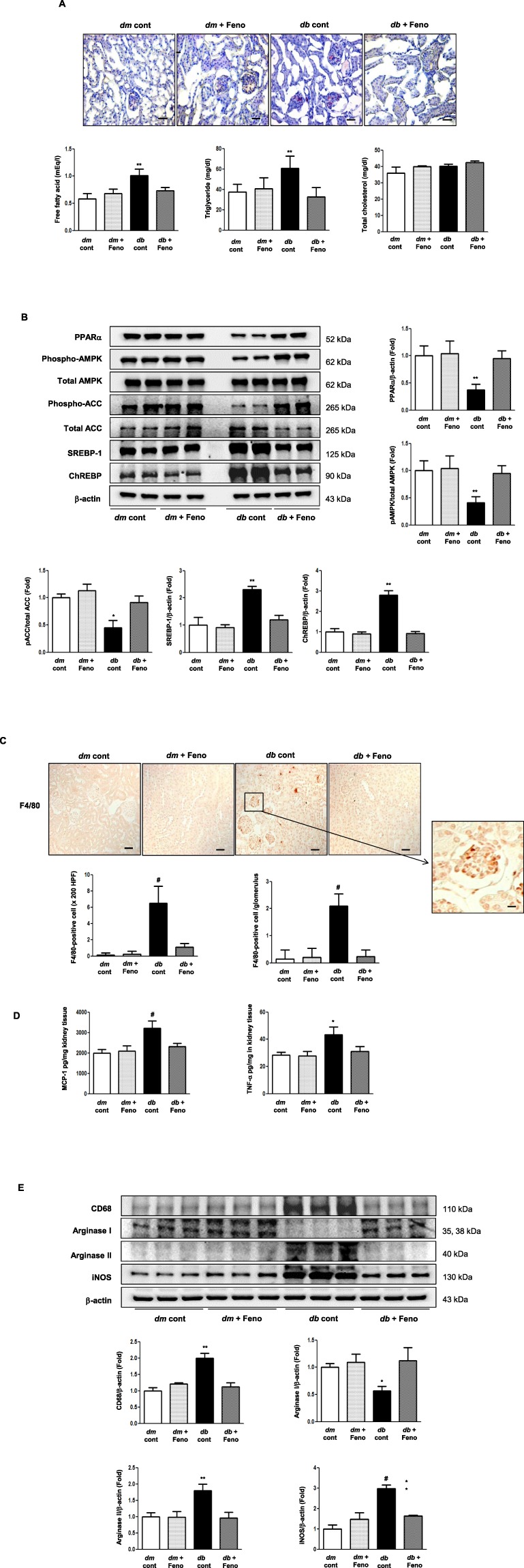
Phospho-AMPK, total AMPK, PPARα, phospho-ACC, total ACC, SREBP-1, ChREBP, and intra-renal lipid levels in the renal cortex of *db/m* and *db/db* mice, with or without fenofibrate treatment. Representative oil-red O staining of the renal cortex, and quantitative analyses of intra-renal NEFA, TG, and TC concentrations (**A**); Scale bars represent 60 µm (x200). Representative western blotting is shown for phospho-AMPK, total AMPK, PPARα, phospho-ACC, total ACC, SREBP-1, ChREBP, and β-actin and their quantitative analyses (**B**). Representative sections with immunohistochemical staining and quantitative analyses for F4/80-positive cells (**C**); Scale bars represent 60 µm (x200). The intrarenal MCP-1 and TNF-α concentrations of the study mice (**D**). Representative western blot analysis of CD68, arginase I, arginase II, iNOS, and β –actin, and their quantitative analyses (**E**). ^*^P < 0.05, ^**^P < 0.01 *vs. db/db* mice. Cont, control; Feno, fenofibrate.

We determined the degree of lipotoxicity by measuring intrarenal contents of NEFA, TG, TC and relevant molecular expression involved in fatty acid synthesis and fatty acid β-oxidation. Oil red O was used to stain neutral TGs and lipids in the renal cortex. Red lipid droplets evenly distributed throughout the renal cortex of the diabetic mice disappeared upon fenofibrate treatment. Fenofibrate ameliorated increases in intrarenal NEFA and TG levels (Fig. 1A). Fenofibrate increased and recovered PPARα, AMPK, and the pACC/total ACC ratio to the level of the non-diabetic controls, while decreasing the expression of SREBP-1 and ChREBP in the diabetic mice (Fig. 1B). Thus, fenofibrate-induced activation of AMPK and PPARα ameliorates intrarenal lipotoxicity through decreased lipid synthesis and increased fatty acid β-oxidation. These changes correlated with decreased inflammation, as evidenced by reduced expression of intrarenal monocyte chemoattractant protein-1 (MCP-1), TNF- α, and number of F4/80-positive cells in the fenofibrate-treated diabetic mice, by 27.8%, 28.3%, and 88.6%, respectively (Fig. 1C,D). Moreover, fenofibrate decreased the expression of CD68, arginase II, and inducible nitric oxide synthase (iNOS) (Fig. 1E), suggesting reduced mononuclear cell, neutrophil, and M1 macrophage infiltrations in association with decreased degree of inflammation in the renal tissue.

### Amelioration of intrarenal inflammation and fibrosis is associated with attenuated lymphatic proliferation in the kidney

**Figure 2:**
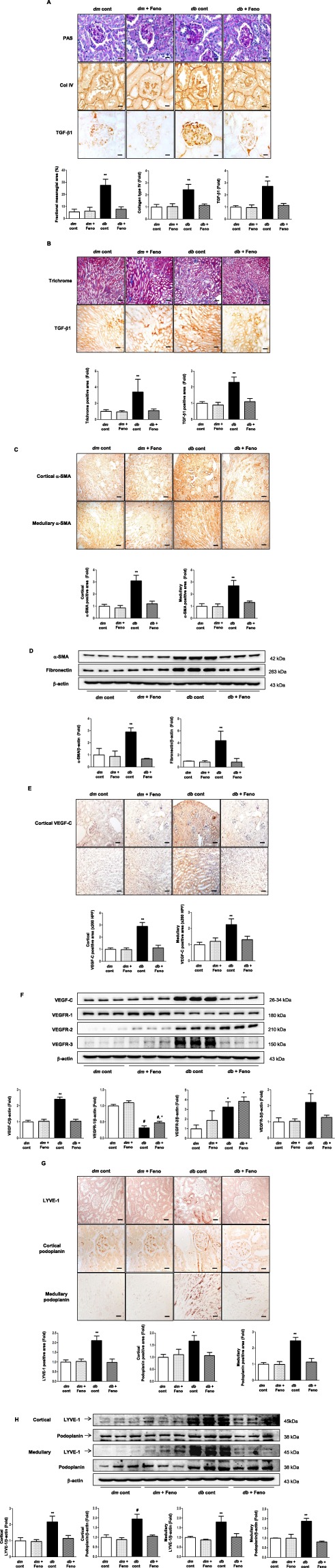
Changes in glomerular and tubular phenotypes in association with fibrosis and lymphangiogenesis in *db/m* and *db/db* mice, with or without fenofibrate treatment. Representative sections stained with PAS reagent are shown to estimate the mesangial fractional area (%), together with a quantitative analysis by groups. Immunohistochemical staining and quantitative analyses for type IV collagen and TGF-β1 (**A**); Scale bars represent 60 µm (x200). Representative sections stained with trichrome and quantitative analysis for trichrome positive area, with immunohistochemical staining and quantitative analyses for TGF-β (**B**); Scale bars represent 60 µm (x200), and α-SMA (**C**); Scale bars represent 120 µm (x100), in the renal cortex and medulla. Representative western blot analysis of α-SMA, fibronectin, and β-actin levels and their quantitative analyses (**D**). Representative immunohistochemical staining for VEGF-C and the quantitative analysis of the results (**E**); Scale bars represent 60 µm (x200). Representative western blot and quantitative analysis of VEGF-C, VEGFR-1, VEGFR-2, VEGFR-3, and β-actin and their quantitative analyses (**F**). Representative immunohistochemical staining for LYVE-1, and podoplanin (**G**); Scale bars represent 60 µm (x200). Representative western blot of LYVE-1, podoplanin, and β-actin and their quantitative analyses (**H**). ^*^P < 0.05 ^**^P < 0.01, and ^#^*P* < 0.001 compared with other groups. Col IV, type IV collagen.

Improvement in renal glomerular phenotype in association with reduced fibrosis was indicated by decreases in the fractional mesangial area and the expression of collagen type IV and TGF-β1 in the fenofibrate-treated groups by 72%, 53.5%, and 58%, respectively (Fig. 2A). The renal tubular phenotype was ameliorated (i.e. decreases in trichrome and TGF-β1 positive areas by 67.6% and 52.2%, respectively) in the treatment group (Fig. 2B). Similarly, extent of cortical and medullary α-SMA positive areas was reduced by 61.3% and 51.5%, respectively and the expression of α-SMA and fibronectin decreased in the diabetic mice treated with fenofibrate (Fig. 2C,D). These renal phenotypical improvements were accompanied by reduced intensities of fibrosis and inflammation in the fenofibrate treatment group. We chose LYVE-1, VEGFR, and a type 1 integral membrane glycoprotein, podoplanin as the LEC markers in the kidney. While normal kidneys exhibit considerable amounts of lymphatics in the renal cortex, its expression is scarce in the medulla^12^; therefore we investigated the expression of VEGF-C and relevant LECs in both regions because both diabetic and non-diabetic diseased kidneys demonstrate copious distribution of lymphatic vessels in the cortex and medulla. Extent of VEGF-C positive area was reduced in the cortex and medulla by 61.4% and 41.3%, respectively (Fig. 2E) and increased expression VEGF-C and VEGFR-3 decreased in diabetic mice with fenofibrate treatment (Fig. 2F). Interestingly, there were no differences in VEGFR-1 and VEGFR-2 expression in *db/db* mice with fenofibrate. Consistently, increased expression of LYVE-1 and podoplanin in the renal cortex and medulla decreased by 53.6%, 35.9%, and 53.5%, respectively in diabetic mice with fenofibrate treatment (Fig. 2G,H). Lymphatic proliferation in the renal cortex and medulla of diabetic mice was attenuated in line with ameliorated intrarenal inflammation and fibrosis as demonstrated by downregulation in the expression of VEGF-C, LYVE-1, podoplanin and VEGFR-3 in the fenofibrate treated group.

### Renal functional parameters and renal cell apoptosis were improved in association with decreased oxidative stress

**Figure 3:**
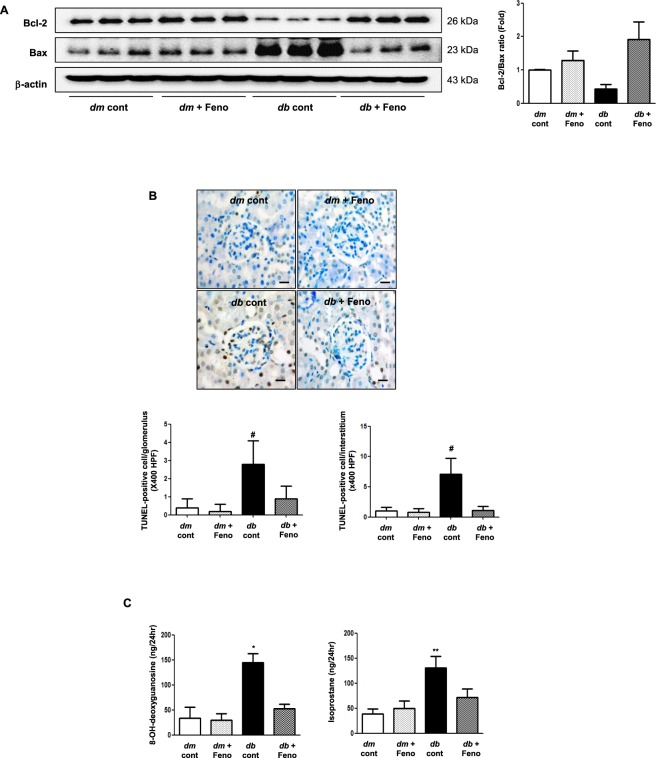
Changes in renal apoptosis and oxidative stress in *db/m* and *db/db* mice, with or without fenofibrate treatment. Representative western blot analysis of Bax, bcl-2, and β-actin, and their quantitative analyses (**A**). Representative pictures of TUNEL-positive cells and their quantitative analyses (**B**); Scale bars represent 60 µm (x200). The 24-h urinary 8-OH-dG and isoprostane concentrations in the study mice (**C**). *P < 0.05, **P < 0.01, and ^#^*P* < 0.001 compared with other groups.

We next verified whether decreased lymphatic proliferation in association with subsequent attenuation in renal inflammation and fibrosis was further linked to renal functional enhancement through reduced burden of systemic oxidative stress and apoptosis. *Db/db* mice exhibited features of diabetes; increased fasting blood sugar and HbA_1c_ levels, body weight, food intake, and urine volume. Albuminuria was reduced in the *db/db* fenofibrate group compared with the *db/db* control group. Serum TC and TG levels decreased with fenofibrate treatment (Table 1). Clinical parameters associated with diabetic condition were relieved in fenofibrate treatment group. Intrarenal apoptosis was attenuated as demonstrated by increases in Bcl-2/Bax expression and decreases in glomerular and interstitial TUNEL-positive cells (Fig. 3A,B). Moreover, oxidative stress markers 8-OH-dG and isoprostane in 24 h urine collection were reduced in the diabetic mice treated with fenofibrate (Fig. 3C). Thus, previously observed intrarenal attenuation of lymphatic sprouting may be linked with reduced apoptosis and oxidative stress in the kidney.

**Table 1:** Biochemical and physical characteristics of study groups.

Characteristics	db/m control	db/m Feno	db/db control	db/db Feno
Body weight (g)	32.1 ± 1.4	29.2 ± 1.8	54.3 ± 4.1^#^	56.6 ± 3.4^#^
Food intake (g/d)	3.3 ± 0.5	3.6 ± 0.7	10.2 ± 2.3^#^	8,9 ± 2.1^#^
FBS (mmol/l)	7.49 ± 0.3	8.21 ± 1.0	30.25 ± 2.7^#^	22.14 ± 1.9^#^, ^&^
HbA1c (%)	4.1 ± 0.4	4.2 ± 0.2	11.7 ± 1.5^#^	8.3 ± 1.0^#^, ^&^
Urine volume (mL)	1.0 ± 0.4	1.1 ± 0.2	17.8 ± 7.5^#^	4.3 ± 1.1^*^,^&^
Serum Cr (μmol/l)	9.72 ± 1.8	10.61 ± 13.3	11.49 ± 7.1	10.61 ± 4.4
24 hr albuminuria (µg/day)	10 ± 4	11 ± 5	277 ± 86^#^	106 ± 22^*^,^&^
Serum TC (mmol/l)	0.93 ± 0.1	1.03 ± 0.0	1.04 ± 0.0^*^	1.10 ± 0.0
Serum TG (mmol/l)	0.42 ± 0.1	0.46 ± 0.1	0.69 ± 0.1^#^	0.37 ± 0.1^#^,^&^
Serum NEFA (mEq/L)	1.10 ± 0.16	1.09 ± 0.18	1.40 ± 0.18	1.24 ± 0.21

FBS, fasting blood sugar; Cr, creatinine; TC, total cholesterol; TG, triglyceride; NEFA, non-esterified fatty acid; Feno, fenofibrate.

^*^P < 0.05 vs. *db/db* mice, ^**^P < 0.01 vs. *db/m* mice, ^#^*P* < 0.001; ^&^*P* < 0.05 vs. *db/db* mice.

### Improvements in renal oxidative stress and functional parameters were associated with attenuated intrarenal lipotoxicity and lymphatic proliferation in other rat models

**Figure 4:**
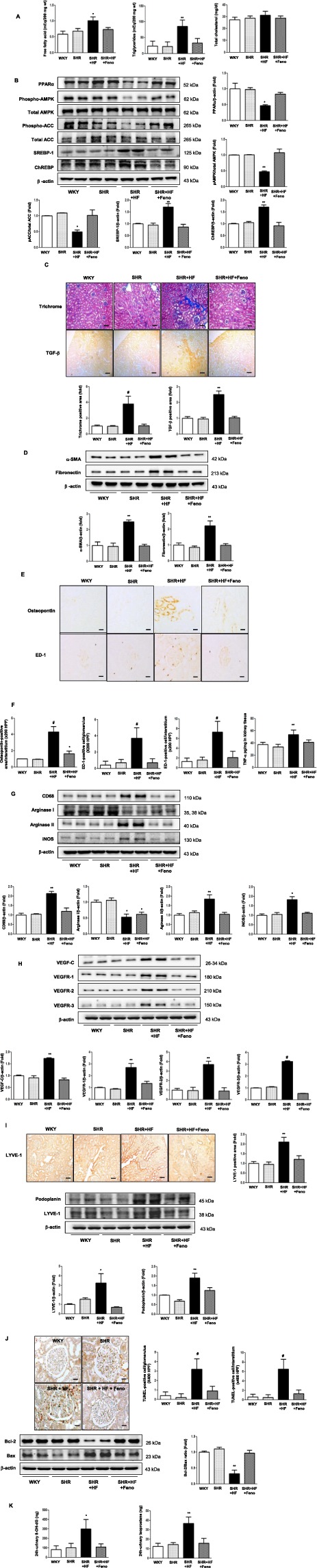
Attenuated renal lipotoxicity improves lymphatic proliferation and renal phenotypes through decreased inflammation, fibrosis, apoptosis and oxidative stress in the renal cortex of HFD SHRs with fenofibrate treatment. Quantitative analyses of intra-renal NEFA, TG, and TC concentrations in the study mice (**A**). Representative western blotting is shown for phospho-AMPK Thr^172^, total AMPK, PPARα, phospho-ACC, total ACC, SREBP-1, ChREBP, and β-actin and their quantitative analyses (**B**). Representative sections stained with trichrome and quantitative analysis for trichrome positive area, with immunohistochemical staining and quantitative analyses for TGF-β in the renal cortex and medulla (**C**); Scale bars represent 60 µm (x200). Representative western blot analysis of α-SMA, fibronectin, and β-actin levels and their quantitative analyses (**D**). Representative sections of immunohistochemical staining for osteopontin and ED-1positive cells and their quantitative analyses (**E**); Scale bars represent 60 µm (x200). Intrarenal TNF-α concentrations of the study mice (**F**). Representative western blot analysis of CD68, arginase I, arginase II, iNOS, and β -actin and their quantitative analyses (**G**). Representative western blot and quantitative analysis of VEGF-C, VEGFR-1, VEGFR-2, VEGFR-3, and β-actin and their quantitative analyses (**H**). Representative immunohistochemical staining for LYVE-1 and the quantitative analysis of the results; Scale bars represent 60 µm (x200). Representative western blot of LYVE-1, podoplanin, and β-actin and their quantitative analyses (**I**). Representative pictures of TUNEL-positive cells and western blot analysis of Bax, bcl-2, and β-actin and their quantitative analyses (**J**); Scale bars represent 60 µm (x200). Quantitative analyses of the 24-h urinary 8-OH-dG and isoprostane concentrations (**K**) in the study mice. ^*^P < 0.05, ^**^P < 0.01, and ^#^*P* < 0.001 compared with other groups. Feno; fenofibrate, HF, high-fat diet.

HFD SHRs manifested features of metabolic syndrome which were improved by fenofibrate; it reduced systolic blood pressure, the amount of albuminuria, and serum TC without affecting HbA_1c_ level. Fenofibrate-induced amelioration of intrarenal NEFA and TG accumulation (Fig. 4A) was accompanied with increased expression of PPARα, pAMPK/total AMPK, pACC/total ACC and decreased expression of SREBP-1, and ChREBP (Fig. 4B). Renal tubulointersitial fibrosis was reduced as represented by trichrome positive area, TGF-β, α-SMA, and fibronectin expression in fenofibrate treated group (i.e. decreases in trichrome and TGF-β1 positive areas by 72.6% and 58.8%, respectively) (Fig. 4C,D). Increased immunostaining for ED-1, osteopontin and expression of TNF-α (Fig. 4E,F) was ameliorated in fenofibrate treated group. (i.e decreases in ED-1 positive cells in glomerulus/interstitium, osteopontin, and TNF-α by 81.1%, 71.2%, 61.9%, and 23%, respectively). ED-1 is an antibody against cellular marker CD68 specific for activated macrophage. Osteopontin is biosynthesized by macrophages and stimulated upon exposure to pro-inflammatory cytokines^13^. Decreased expression of CD68, arginase II, and iNOS and increased expression of arginase I level were observed in the same group (Fig. 4G). Lymphatic proliferation was ameliorated (i.e. decreased expression of VEGF-C, VEGFR-1,VEGFR-2, VEGFR-3, LYVE-1, and podoplanin) (Fig. 4H,I) in line with improved renal apoptosis (i.e. decreased TUNEL-positive cells in glomerulus and interstitium by 80% and 71.9%, respectively and increased expression of Bcl-2/Bax ratio) (Fig. 4J) and oxidative stress (i.e. decreased urine 8-OH-dG and isoprostane levels) (Fig. 4K) in fenofibrate treated HFD SHRs. It is demonstrated that macrophage may play a pivotal role in regulating lymphangiogenesis in the context of intrarenal inflammation and lipotoxicity in other rat models that was independent of systemic glucose level.

### Amelioration of intracellular lipotoxicity reduces the expression of lymphangiogenic markers in HK-2 and RAW 264.7 cells grown in palmitate and high-glucose media in association with M1 macrophage inactivation

**Figure 5:**
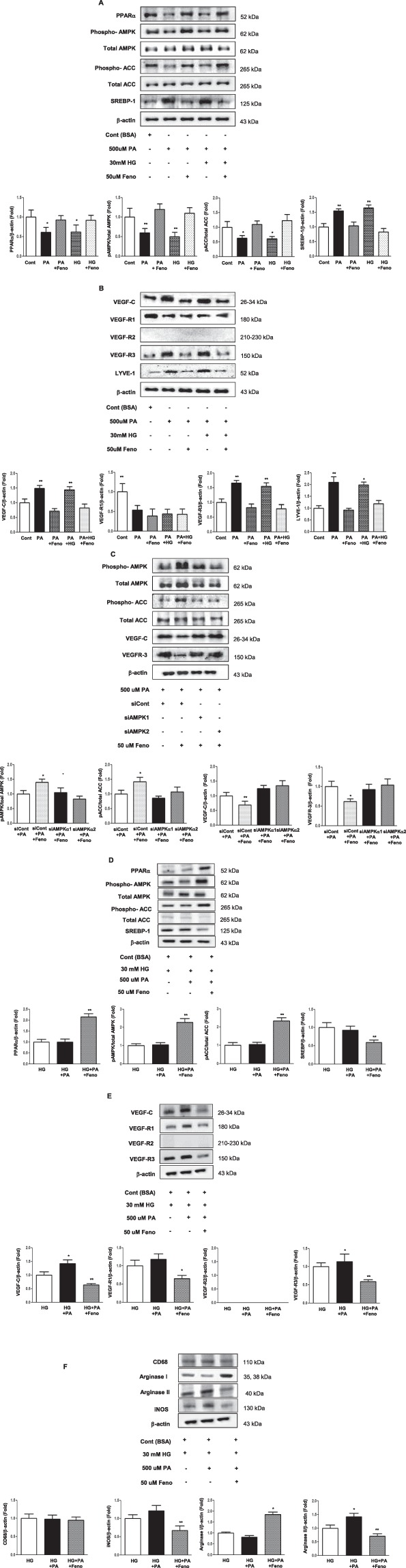
The effect of fenofibrate on intracellular signaling and lymphangiogenesis in cultured HK-2 cells and RAW 264.7 cells cultured in palmitate and/or high glucose. Representative western blotting analysis of Phospho-AMPK, total AMPK, PPARα, phospho-ACC, total ACC, SREBP-1, and β-actin levels, and their quantitative analyses (**A,D**). Representative western blotting analysis of VEGF-C, VEGFR-1, VEGFR-2, VEGFR-3, LYVE-1, and β-actin, and their quantitative analyses (**B,E**). Representative western blotting analysis of CD68, arginase I, arginase II, iNOS, and β-actin levels, and their quantitative analyses (**F**). The effect *Ampkα1* and *Ampkα2* siRNAs on the fenofibrate-stimulated AMPK-pACC signaling and on the expression of VEGF-C and VEGFR-3 in HK-2 cells cultured in palmitate. Representative western blotting analysis of Phospho-AMPK, total AMPK, phospho-ACC, total ACC, VEGF-C, VEGFR-3, and β-actin levels, and their quantitative analyses (**C**). ^*^P < 0.05 and ^**^P < 0.01 compared to other groups. Feno; fenofibrate, HG, high glucose; LG, low glucose; PA, palmitate.

Because *in vivo* study demonstrated expression of VEGF-C, LYVE-1 and podoplanin most prominent within tubules and also because lymphangiogenic capacity of macrophages is well established in state of inflammation, we investigated the relationship between lipotoxicity and lymphangiogenesis *in vitro* by using HK-2 and RAW264.7 cells. HK-2 and RAW264.7 cells grown in either palmitate or palmitate plus high-glucose medium were treated by fenofibrate. Expression of PPARα, pAMPK/total AMPK, pACC/total ACC increased and that of SREBP-1decreased in fenofibrate-treated cells grown in both media (Fig. 5A,D) and expression of VEGF-C, VEGFR-3, and LYVE-1 decreased in the same group (Fig. 5B,E). Consistently, expression of iNOS and arginase II decreased in fenofibrate treated macrophages grown in palmitate and high-glucose medium, signifying involvement of pro-inflammatory M1 macrophage action in association with its lymphangiogenic capacity (Fig. 5F). Next, fenofibrate was used to stimulate siRNAs for *Ampkα1* and *Ampkα2* in cultured HK-2 cells grown in palmitate media. Fenofibrate activated AMPK–pACC signaling and reduced the expression of VEGF-C and VEGFR-3 in palmitate conditions. In contrast, transfection with *Ampkα1* and *Ampkα2* siRNAs suppressed fenofibrate-induced AMPK–pACC signaling and increased the expression of VEGF-C and VEGFR-3 (Fig. 5C). These results demonstrated the lymphangiogenic capacity of PTECs and macrophages under lipotoxic condition and confirmed that reduced lymphangiogenesis was associated with inhibition of pro-inflammatory action of M1 macrophage in line with decreased lipotoxicity via lipid-lowering action of PPARα agonist fenofibrate that is AMPK dependent.

## Discussion

Recent studies show that human renal biopsy specimens of various renal diseases, including DN, revealed increased numbers of lymphatic vessels at sites of tubulointerstitial lesions, which correlated with the degree of tissue damage as determined by fibrosis and inflammation^4^. Addressing to this concept of chronic inflammation and fibrosis with regard to lymphatics, we presumed that lymphangiogenesis might be prevalent in DN and hypothesized that lymphatic proliferation may waver in the context of improved lipotoxicity in the kidneys. Herein, we used fenofibrate to construct an environment in which diabetes- and high-fat diet-induced lipotoxicity can be alleviated through the activation of PPARα and AMPK. PPARα activation is associated with an increase in fatty acid catabolism and subsequent ATP production, which, combined with decreased cytotoxic fatty acid peroxidation, promotes cell viability^14^. Furthermore, AMPK activation inhibits lipogenesis and enhances fatty acid oxidation through targets such as SREBP-1 and ACC^15^, which is consistent with our current findings.

Lymphatic proliferation was prevalent in the renal cortex and medulla of diabetic mice and HFD SHRs as demonstrated by increased expression of VEGF-C, podoplanin, LYVE-1, and VEGF-R3. Some isoforms of podoplanin are expressed predominantly in lymphatic tissues and are activated in the pro-lymphangiogenic state to promote the formation of connecting lymphatics by facilitating LEC adhesion and migration^16^. Due to the intrinsic nature of podoplanin in constituting the shape of the podocyte, a fair amount of podoplanin is stained around the glomeruli of both non-diabetic and diabetic mice. However, this contrast is enhanced as diabetic mice show a larger amount of podoplanin in the medulla as well, which signifies the development of lymphatic proliferation. The CD44 homologous hyaluronic acid-binding protein LYVE-1 is expressed specifically by LECs and might contribute to promoting pathological lymphangiogenesis in injured tissues, because LYVE-1 cleavage from LECs occurs in lymphatic vessels undergoing chronic inflammation in response to VEGF-A^17^. LECs express various VEGFRs, among which VEGFR-3 shows the highest affinity toward VEGF-C, signifying the presence of lymphangiogenesis^18–20^. Previously, Sakamoto *et al*. demonstrated increased VEGF-C expression in PTECs, suggesting its important role as a source of VEGF-C in renal diseases^4^. Thus, to address lipotoxicity associated with lymphangiogenic potential of PTEC, we carried out *in vitro* experiment using HK-2 cells grown in palmitate and high-glucose media. Fenofibrate prevented palmitate- and high glucose-induced expression of VEGF-C, VEGFR-3, and LYVE-1 which was accompanied by activation of PPARα-AMPK-pACC signaling and suppression of SREBP-1 and ChREBP, suggesting attenuated lymphatic proliferation is associated with improved lipid metabolism.

Apart from the impact of glucose per se and/or interleukin-1β and TNF-α, the pivotal role of macrophages in the development of lymphangiogenesis is speculated in inflammatory diabetic conditions^21^. Macrophage contributes to lymphatic sprouting by paracrine excretion of VEGF-C and by transdifferentiating into LECs^22–24^. We explored which subpopulation of macrophage was responsible for the activation of lymphatic growth; while the expression of intrarenal CD68, arginase II, and iNOS increased, that of arginase I decreased in diabetic mice and HFD SHRs. Arginase isozyme I and II are markers to distinguish M2 and M1 macrophage phenotypes, respectively. While M1 macrophages are pro-inflammatory, cytotoxic killer cells, up-regulating such genes as iNOS, M2 macrophages produce anti-inflammatory cytokines, typically of VEGF and TGF-β, and participate in the resolution of inflammation^25^. Enhanced expression of M1 to a greater extent than that of M2 with concomitant increases in the expression of TGF-β, TNF-α and VEGF-C signify the presence of an on-going strong immunological response in the setting of lymphangiogenesis that is pro-inflammatory. Studies investigating pro-lymphangeogenic capacity of macrophage in unilateral ureteral obstruction (UUO) mice model demonstrated M2 phenotype to be the main source of lymphatic endothelial progenitor cells. This is distinguished from our finding that primarily involves M1-like macrophage actions. We assume this discrepancy may be ascribable to distinct nature of underlying diseases; while pro-angiogenic M2-like macrophages congregate in areas of hypoxia due to UUO, pro-inflammatory M1 phenotype predominate in regions of tissue inflammation.

Previous literatures investigating the role of VEGF-C in lymphangiogenesis have been contradictory; while up-regulation of VEGF-C with a link to connective tissue growth factor has been demonstrated in fibrosis-associated renal lymphagiogenesis^26^, local treatment with VEGF-C restored lymphatic function in the skin of apoE^−/−^ mice^8^. Does increased expression of VEGF-C signify presence of normal functioning lymphatics? Quite the contrary; the current study demonstrated increased extent of lymphatic proliferation (i.e. increased expression of VEGF-C) in diabetic mice that correlated with extensive α-SMA, trichrome, and TGF-β1 positive areas, and with irregular, discontinuous type IV collagen-positive basement membranes. From these results, we carefully speculate that a balance between TGF-β and VEGF-C needs be maintained for the growth of normal functioning lymphatics. TGF-β induced up-regulation of VEGF-C in the inflammatory condition is expected to facilitate removal of toxic metabolites. However, in DN, which denotes a state of chronic pro-inflammation, over-sprouting lymphatics due to chronic up-regulation of VEGF-C end up being incomplete and malfunctioning^27^.

In conclusion, we demonstrated evidence that fenofibrate-induced amelioration in intrarenal lipotoxicity and subsequent macrophage waning were accompanied by lymphatic growth attenuation that was in parallel with the extent of renal fibrosis and apoptosis. In this process, removal of intrarenal inflammatory cells and toxic lipid metabolites may have been facilitated through restoration of functioning lymphatics thereby further accomplishing renal functional and phenotypic improvement. These results suggest a causal relationship between lipotoxicity and lymphatic proliferation with a cellular link to macrophage activation, such that pro-inflammatory M1 type macrophages in DN contributed to the development of intrarenal lymphangiogenesis through stimulation of VEGF-C and by its transdifferentiation into LECs that integrate into the sprouting lymphatics. We emphasize the implication of lymphatic vessels in view of PTECs and pro-inflammatory macrophage activation under lipotoxic condition and that dysfunctional lymphatic proliferation can be attenuated by reduced lipotoxicity through PPARα-AMPK pathway, suggesting its potential as a target for lipid-related diseases, including diabetes, especially type 2 diabetes, and HFD-induced renal damage.

## Materials and Methods

### Experimental methods

Male 8-week-old C57BLKS/J *db/m* and *db/db* mice were purchased from Jackson Laboratories (Bar Harbor, ME, USA). Fenofibrate (0.1%, w/w, Sigma, St Louis, MO, USA) was mixed into a standard chow and provided to *db/db* mice (n = 8) and age and gender-matched *db/m* mice (n = 8) for 12 weeks. Control *db/db* (n = 6) and *db/m* mice (n = 6) were fed normal chow. Fenofibrate was administered 125–150 mg kg^−1^ day^−1^ to the treatment groups. 7-week-old male SHRs (SLC, Nakaizu, Japan) and Wistar–Kyoto (WKY) rats were fed either a normal-fat (WKY and SHR, n = 6) or a HFD (SHR + HF, n = 8) for 12 weeks. The SHR-HF rats were treated with fenofibrate (SHR + HF Feno; 20 mg/kg/day in the chow, n = 8) at 8 weeks of age for 12 weeks. The mice were housed in metabolic cages (Nalgene, Rochester, NY) to measure 24-h urinary protein, and were sacrificed by intraperitoneal injection of Rompun 10 mg/kg (Bayer Korea, Ansan, Gyeonggi-Do, Korea) and Zoletil 30 mg/kg (Virbac, Carros, France) at week 20. The dissected kidneys were stored in buffered formalin (10%) for immunohistochemistry. Blood samples after overnight fasting were collected from the left ventricle and stored at −70 °C. An Accu-check meter (Roche Diagnostics, St Louis, Mo) measured the fasting blood glucose. The HbA_1c_ was determined by HPLC (Bio-Rad, Richmond, CA). An auto-analyzer (Wako, Osaka, Japan) measured the total cholesterol (TC), triglycerides (TG), and NEFA concentrations. The experimental protocol was approved by the Institutional Animal Care and Use Committee at the College of Medicine, the Catholic University of Korea (CUMC-2012-0118-02). All methods were carried out in accordance with relevant guidelines and regulations.

### Assessment of renal function, oxidative stress, and intra-renal lipids

Plasma and urine creatinine concentrations were measured by HPLC (Beckman Instruments, Fullerton, CA) and urinary albumin concentration, by (Bayer, Elkhart, IN). Oxidative stress was measured by 8-hydroxy-deoxyguanosine (8-OH-dG) and 24-h urinary 8-epi-prostaglandin F2a′ (isoprostane, OxisResearch, Foster City, CA) levels. Kidney lipids were extracted by Bligh and Dyer method (Waco, Osaka, Japan)^28^.

### Light microscopy study

Kidney samples fixed in 10% buffered formalin and embedded in paraffin were stained with hematoxylin-eosin, periodic acid-Schiff (PAS), and trichrome. The mesangial matrix and glomerular tuft areas were quantified for each glomerular cross-section. More than 30 glomeruli, cut through the vascular pole, were counted per kidney and the average was used for analysis.

### Immunohistochemistry

We performed immunohistochemistry for TGF-β1, type IV collagen, and cell surface glycoprotein F4/80, VEGF-C, LYVE-1, podoplanin, α-smooth muscle actin (α-SMA), ED-1, and osteopontin. Renal tissue sections (4-µm-thick) were incubated overnight with anti-TGF-β1 (1:100; R&D Systems, Minneapolis, MN), anti-COL IV (1:200; Biodesign International, Saco, ME), anti-F4/80 (1:200; Serotec, Oxford, UK), ED-1 (1:1000; Serotec, Raleigh, NC), and osteopontin (1:2000; MPIIIB10, the Developmental Studies Hybridoma Bank, University of Iowa, Iowa, IA) antibodies in a humidified chamber at 4 °C. The antibodies were localized using a peroxidase-conjugated secondary antibody and the Vector Impress kit (Vector Laboratories, Burlingame, CA) with a 3,3-diamninobenzidine substrate solution with nickel chloride enhancement. The sections were dehydrated in ethanol, cleared in xylene, mounted without counterstaining and then were examined in a blinded manner under light microscopy (Olympus BX-50, Olympus Optical, Tokyo, Japan). 20 views (200 × and 400 × magnifications), located randomly in the renal cortex and corticomedullary junction of each slide (Scion Image Beta 4.0.2, Frederick, MD) were quantified. Apoptotic cells in the formalin-fixed, paraffin-embedded tissue were detected by *in situ* TUNEL, using an ApopTag *In Situ* Apoptosis Detection Kit (Chemicon-Millipore, Billerica, MA) and were assessed in the whole glomeruli biopsy under 400 × magnifications.

### Western blotting analysis and enzyme activity determination

Total proteins of the renal cortical tissues were extracted using a Pro-Prep Protein Extraction Solution (Intron Biotechnology, Gyeonggi-Do, Korea). Western blotting was carried out for PPARα, total AMPK, phosphorylated (phospho)-Thr^172^ AMPK, SREBP-1, Carbohydrate regulatory element-binding protein 1 (ChREBP-1), B cell leukemia/lymphoma 2 (BCL-2), BCL-2-associated X protein (BAX), iNOS, CD68, arginase I, and arginase II. Protein concentrations were determined using the Bradford reagent (Bio-Rad., Hercules, CA) (Supplementary file S1).

### Cell culture and small interfering RNA transfection

HK-2 and RAW 264.7 cells grown in 30 mmol/l d-glucose and palmitate (500 μg/ml) were used between passages 25 and 30 and transferred to six-well culture plates at a density of 56,104 cells per well. After reaching 60% confluence, these cells were maintained in a quiescent state in RPMI with 0.1% fetal bovine serum for 48-h and were transfected with 50 nmol/l control siRNA, or 50 nmol/l Ampkα1, and Ampkα2 siRNAs using G-fectin (Genolution Pharmaceuticals, Seoul, Korea). HK-2 cells were then exposed to high glucose, palmitate with or without an additional 24-h application of fenofibrate (50 μg/ml).

### Statistical analysis

The data are expressed as the mean ± standard deviation (SD). Differences between the groups were evaluated using ANOVA with Bonferroni correction by SPSS version 19.0 (SPSS, Chicago, IL, USA). A *P* value of 0.05 was considered statistically significant.

## Supplementary information

Dataset 1
