# Supplementary material for: Attenuated Lymphatic Proliferation Ameliorates Diabetic Nephropathy and High-Fat Diet-Induced Renal Lipotoxicity
Source: Sci Rep. 2019 Feb 13;9:1994. doi: 10.1038/s41598-018-38250-7 (PMC6374395; doi:10.1038/s41598-018-38250-7)

Attenuated Lymphatic Proliferation Ameliorates Diabetic Nephropathy and High-Fat Diet-Induced Renal Lipotoxicity

Yaeni Kim[1], Seun Deuk Hwang[2], Ji Hee Lim[1,3], Min Young Kim[1,3], Eun Nim Kim[1,3], Bum Soon Choi[1,3], Yong-Soo Kim[1], Hye Won Kim[4], and Cheol Whee Park[1,3]

Supplement Figures

Figure 1B

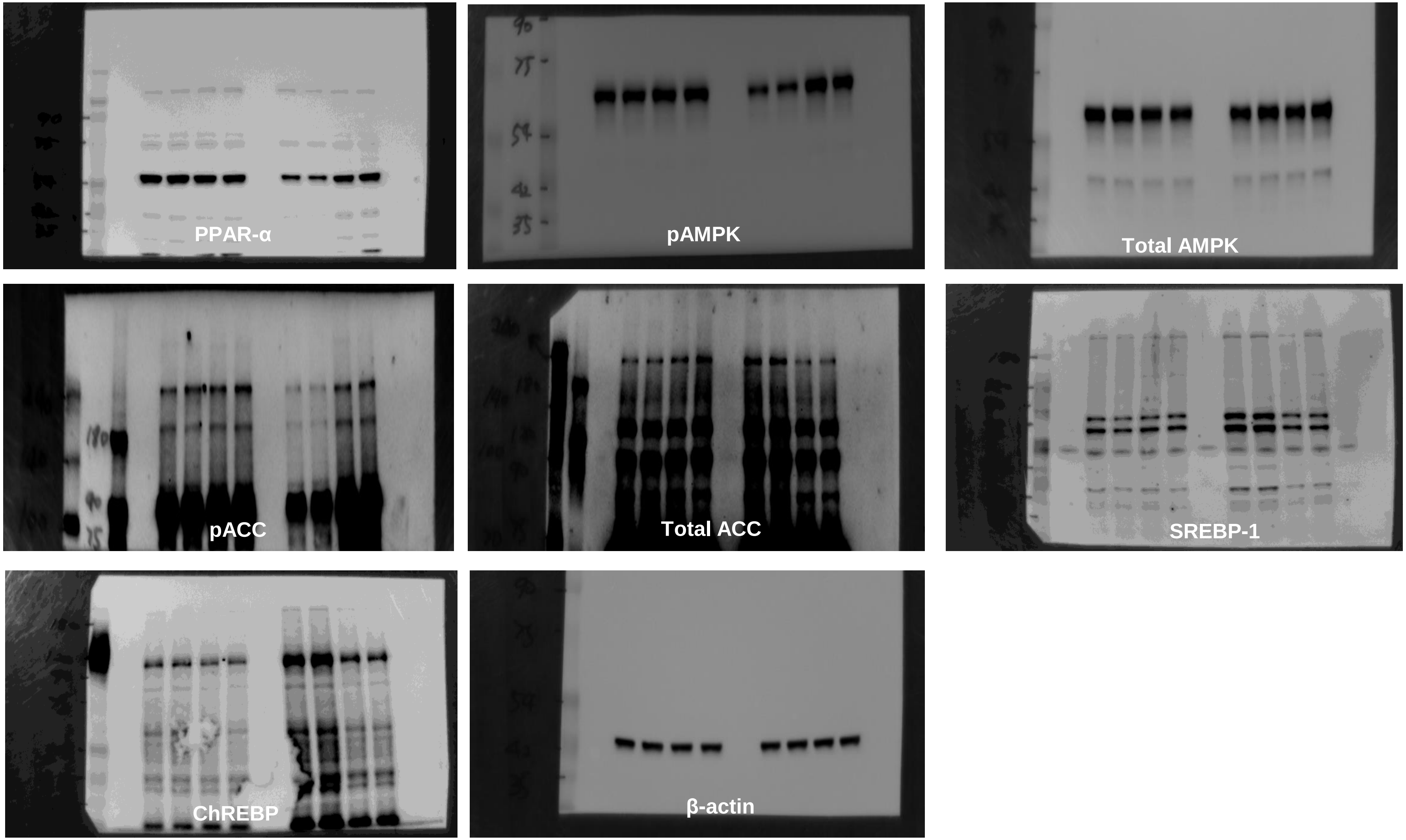

Figure 1E

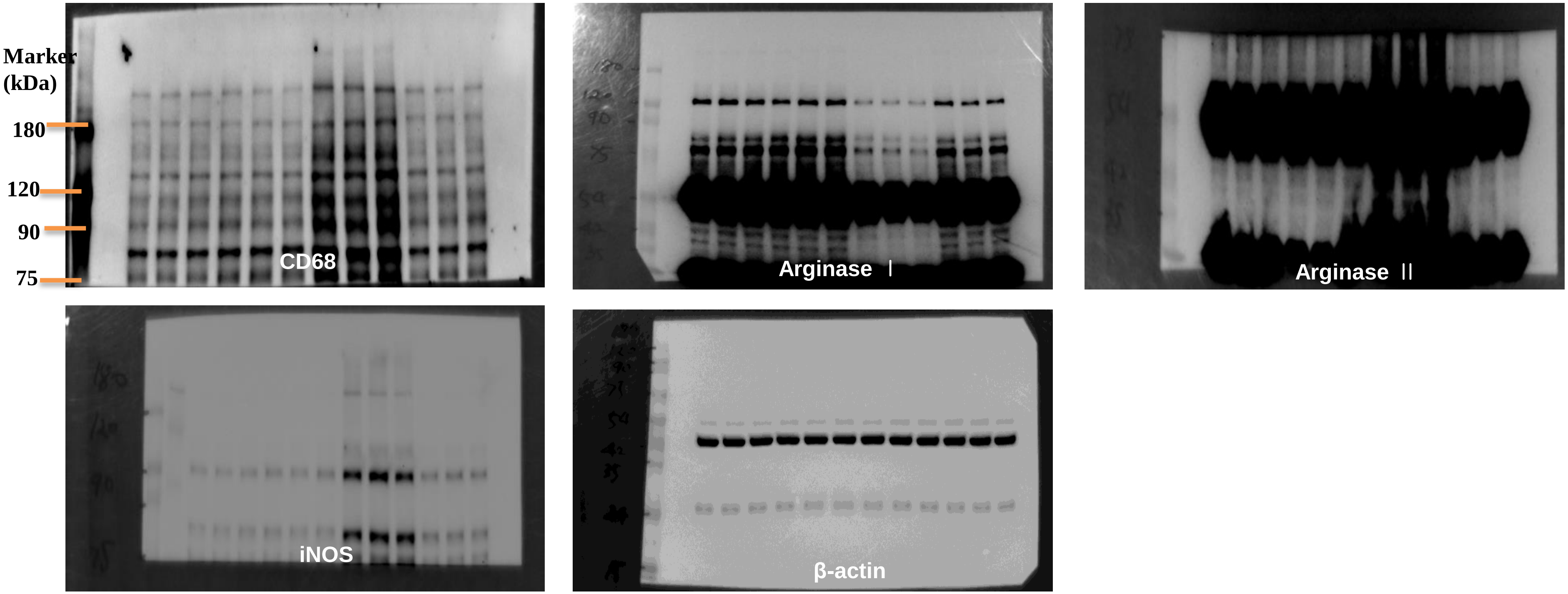

Figure 2D

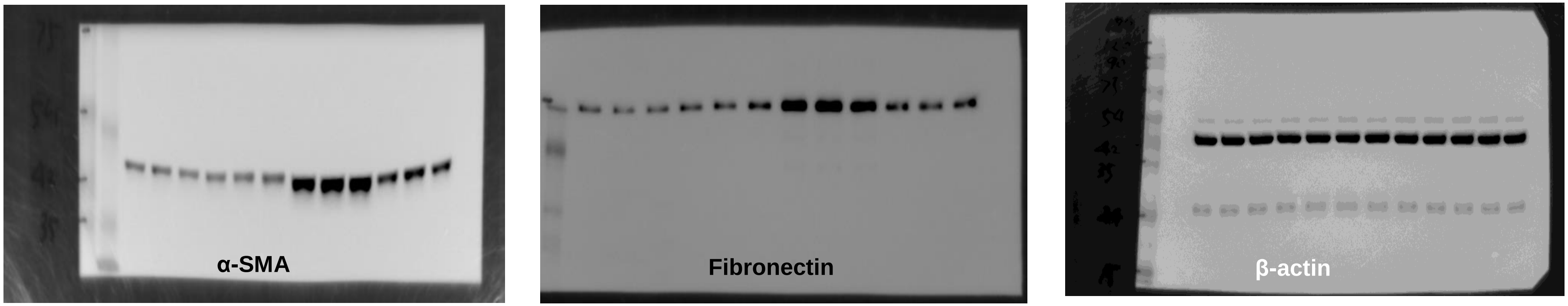

Figure 2F

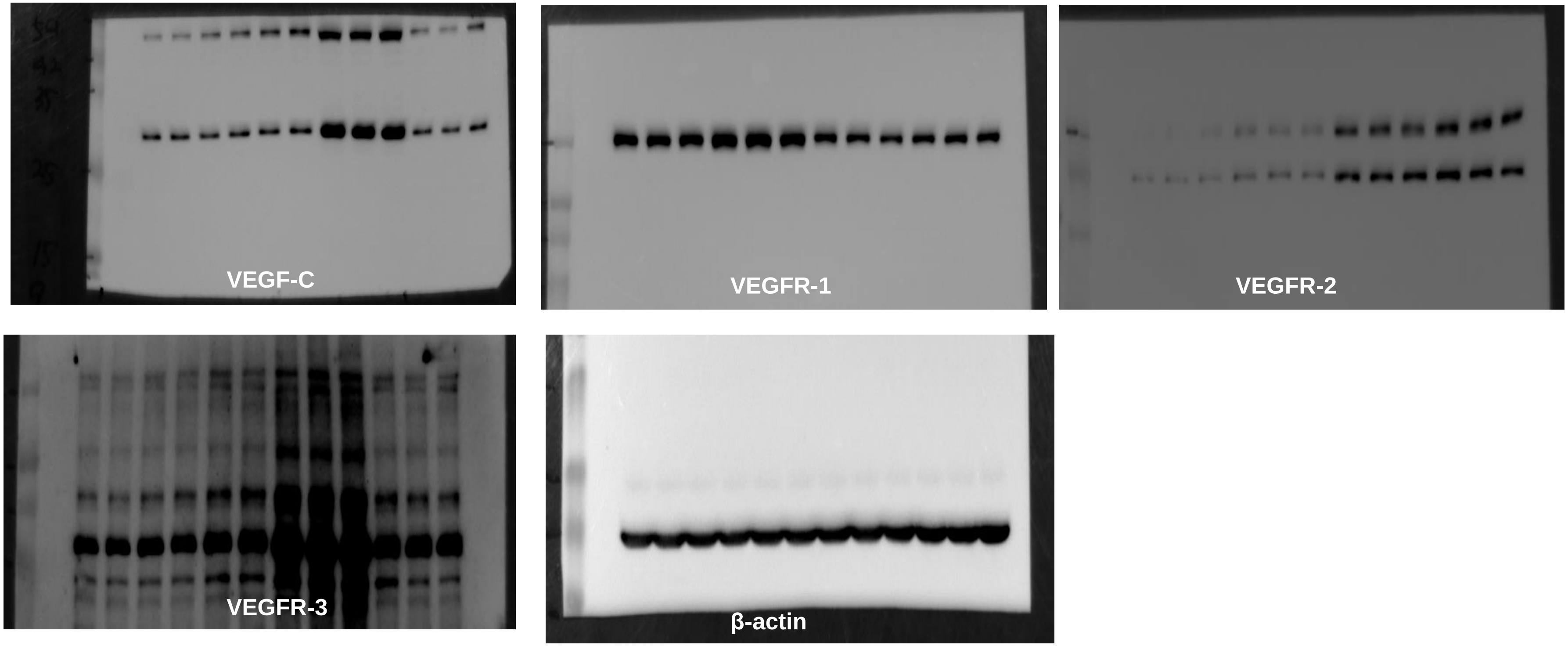

Figure 2H

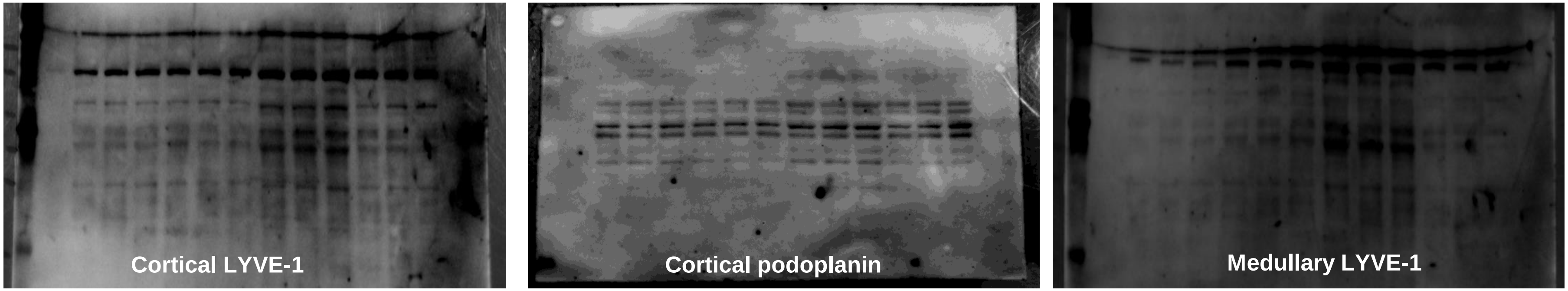

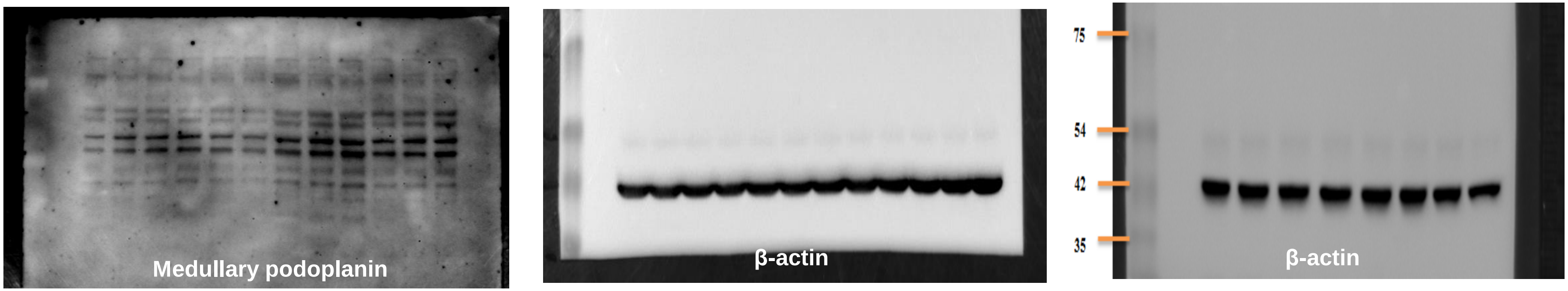

Figure 3A

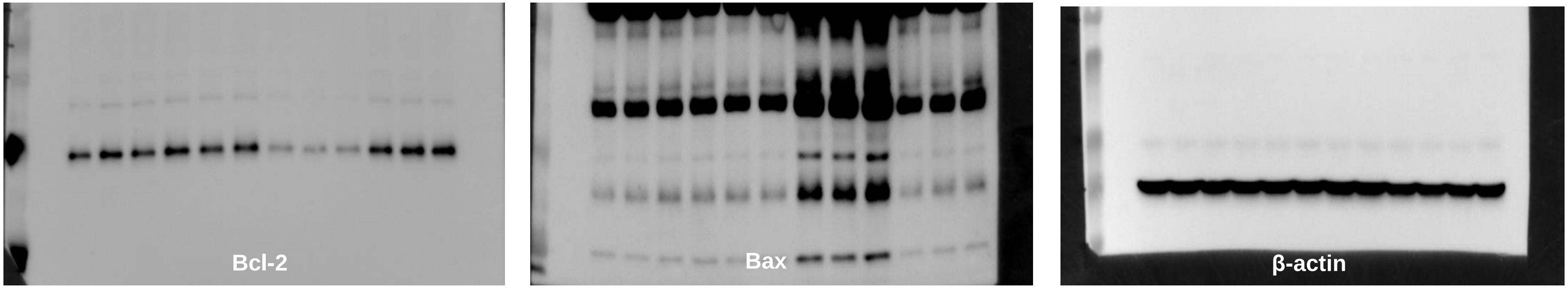

Figure 4B

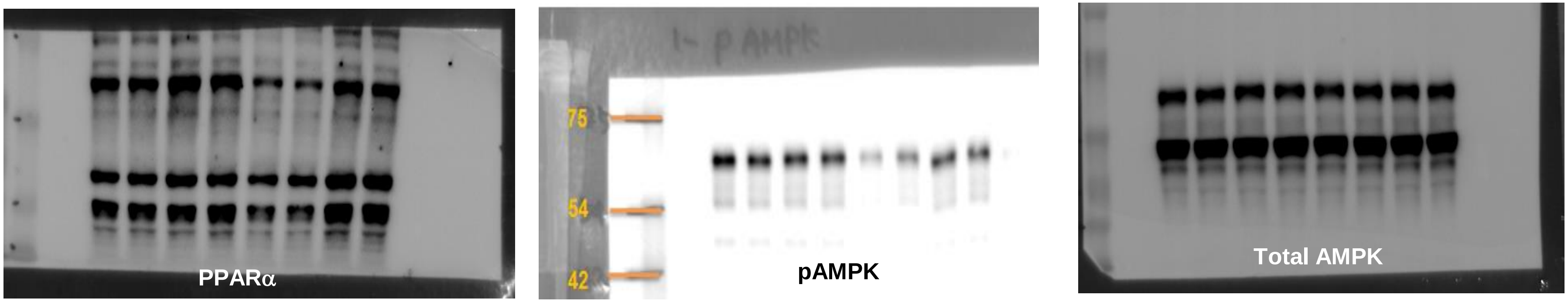

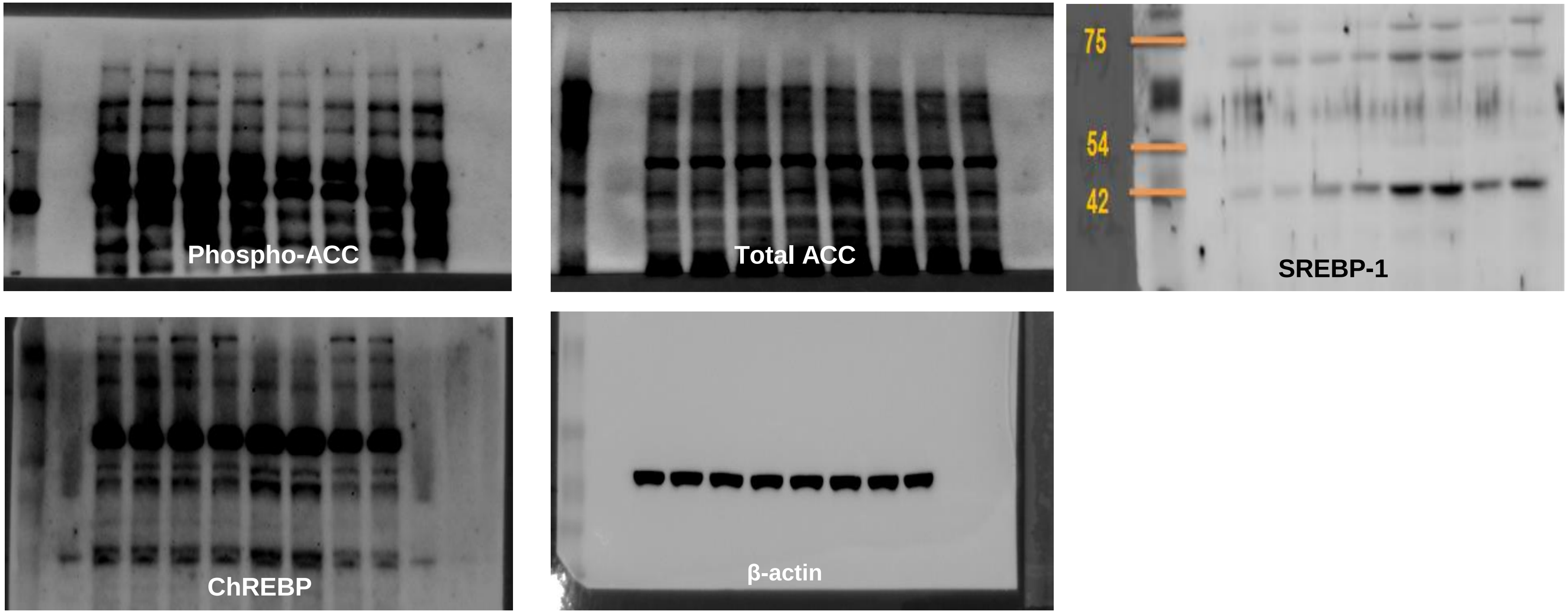
Phospho-ACC
Total ACC
75
54
42
SREBP-1
ChREBP
β-actin

Figure 4D

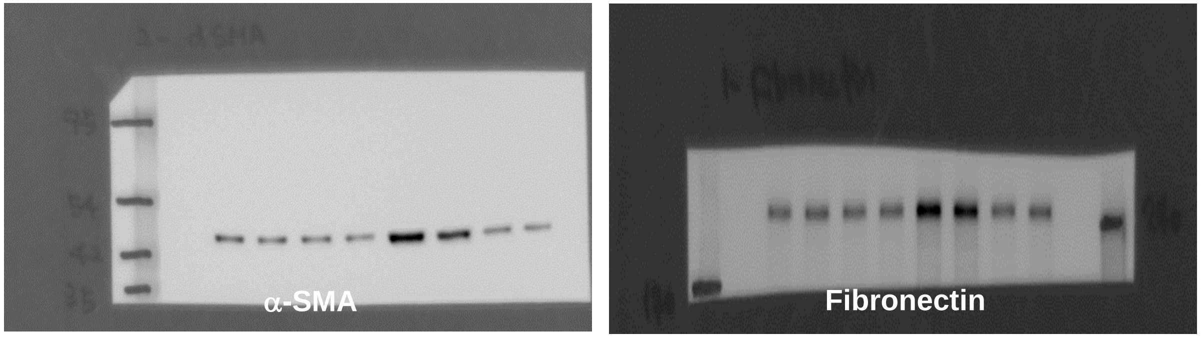

Figure 4G

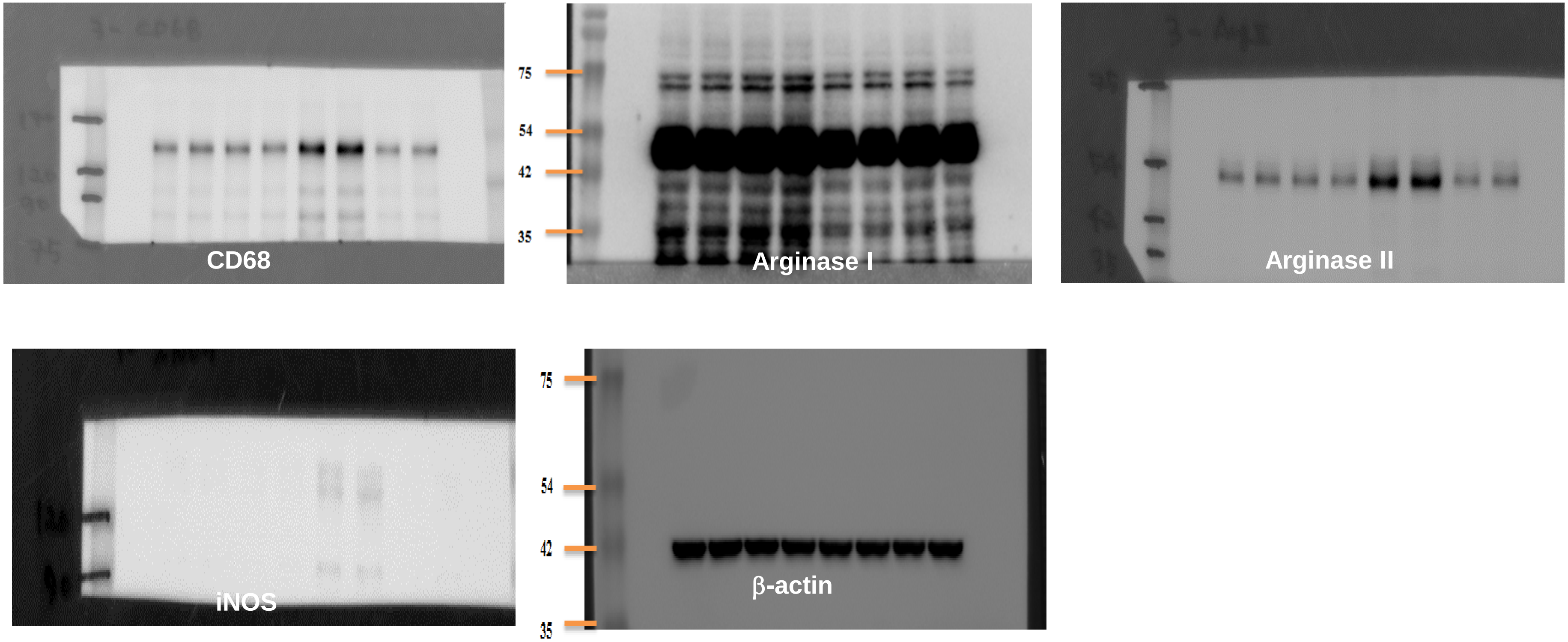

Figure 4H

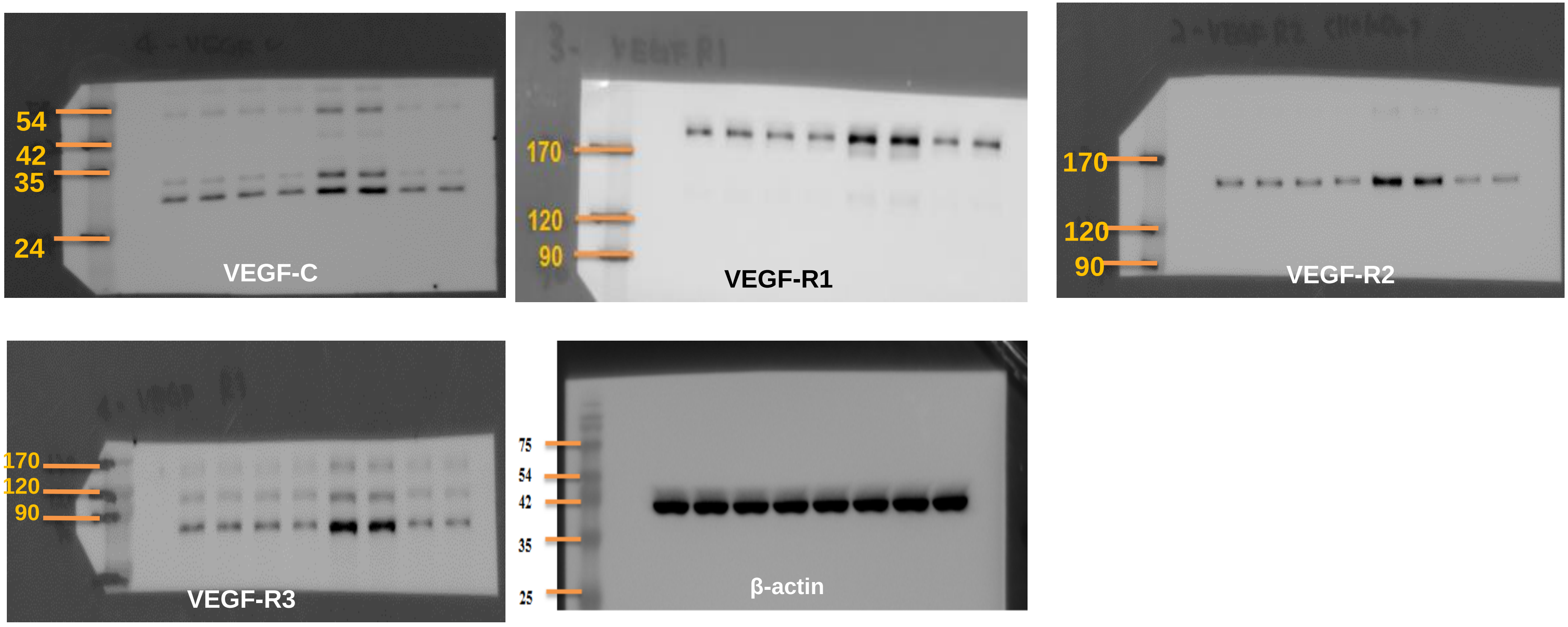

Figure 4i

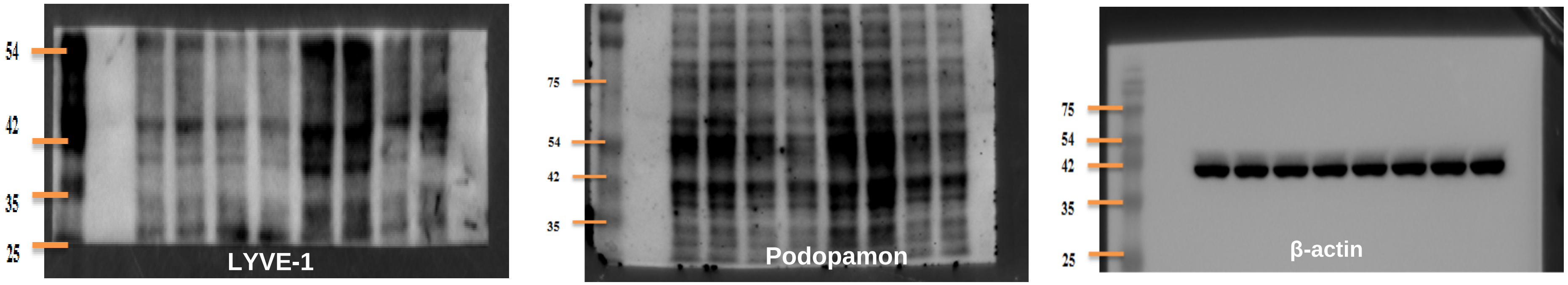

Figure 4J

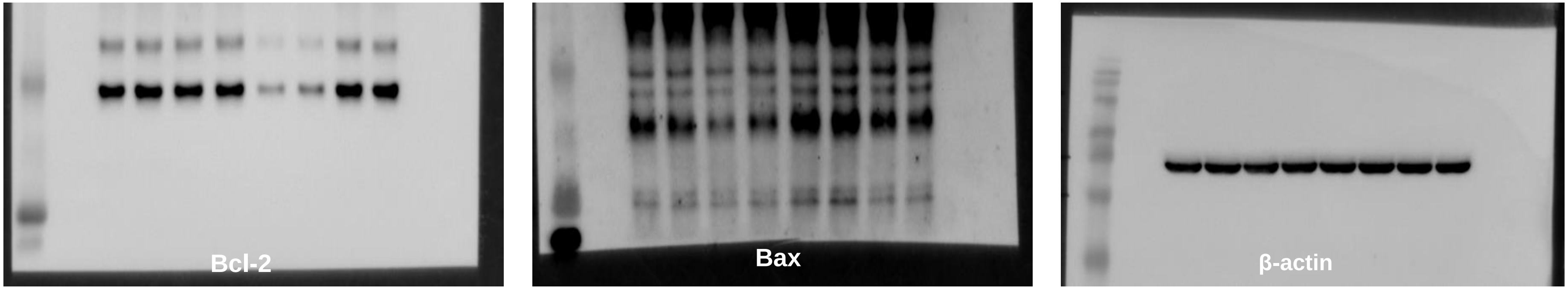

Supplement: Supplementary file 1 — Dataset 1 [file 41598_2018_38250_MOESM1_ESM.pdf]
